# Gingerenone A attenuates diabetic vascular remodeling through AMPK/mTOR/S6K1 signaling

**DOI:** 10.3389/fphar.2026.1706103

**Published:** 2026-01-21

**Authors:** Meixian Chen, Daqian Gu, Yi Lin, Junwei Zhou, Wansheng Lin, Zhi Chen, Zhurong Luo, Pin Chen

**Affiliations:** 1 Fuzong Clinical Medical College of Fujian Medical University, 900th Hospital of PLA Joint Logistic Support Force, Fuzhou, Fujian, China; 2 Department of Critical Care Medicine, Jinling Hospital, Affiliated Hospital of Medical School, Nanjing University, Nanjing, Jiangsu, China; 3 Department of Cardiology, Fuzong Teaching Hospital of Fujian University of Traditional Chinese Medicine, Fuzhou, Fujian, China

**Keywords:** AMP-activated protein kinase (AMPK), diabetic angiopathies, gingerenone A, neointimal hyperplasia, oxidative stress, pharmacokinetics

## Abstract

Diabetes accelerates vascular remodeling and contributes to restenosis after revascularization, in part through oxidative stress-driven dysfunction of vascular smooth muscle cells (VSMCs). Gingerenone A (Gin A), a ginger-derived metabolite with reported metabolic activity, has not been examined in the setting of diabetic vascular remodeling. Here we evaluated whether Gin A mitigates high glucose (HG)-induced VSMC dysfunction and neointimal hyperplasia, with emphasis on redox regulation and AMP-activated protein kinase (AMPK)/mechanistic target of rapamycin (mTOR)/p70 ribosomal S6 kinase 1 (S6K1) signaling. In A10 VSMCs exposed to HG, Gin A reduced proliferation and migration and improved redox status. This was reflected by lower intracellular reactive oxygen species (ROS) and malondialdehyde (MDA), reduced NADPH oxidase 4 (NOX4) expression, and partial restoration of total antioxidant capacity and superoxide dismutase activity. Gin A increased AMPK phosphorylation while suppressing mTOR/S6K1 activation under HG stimulation. AMPK dependence was supported by two perturbation approaches. Compound C attenuated the antiproliferative and anti-migratory effects of Gin A and diminished its effects on mTOR/S6K1 signaling. Additionally, siRNA-mediated knockdown of AMPKα in primary human aortic smooth muscle cells (HASMCs) attenuated the antiproliferative effect of Gin A and blunted the Gin A-associated increase in phosphorylated AMPK. In primary HASMCs, iso-osmotic L-glucose and D-mannitol controls did not reproduce the HG-induced proliferative phenotype, indicating glucose-specific stimulation. In a diabetic rat carotid balloon injury model, oral Gin A attenuated neointimal hyperplasia (reduced intima-to-media ratio), reduced vascular proliferation markers, improved redox indices (lower MDA and higher total antioxidant capacity), and enhanced arterial AMPK activation. In pharmacokinetic studies, a single 10 mg/kg oral dose yielded a maximum plasma concentration (C_max_) of 0.0500 ± 0.0041 μg/mL, a time to C_max_ (T_max_) of 0.29 ± 0.10 h and a terminal half-life of 12.41 ± 4.82 h. A 14-day repeat-dose study revealed no overt biochemical or histopathological toxicity under the tested conditions. Together, these data suggest that Gin A limits diabetic neointimal hyperplasia and VSMC dysfunction through an AMPK-dependent mechanism converging on mTOR/S6K1, accompanied by improved redox homeostasis, and support further evaluation of exposure-response relationships and longer-term safety.

## 1 Introduction

Diabetes mellitus (DM) is a major global health concern, affecting more than 500 million individuals worldwide, with its prevalence projected to exceed 12% by 2045 (Sun et al., 2022). Cardiovascular disease (CVD) remains the leading cause of mortality in patients with diabetes (Ling et al., 2020; Dal Canto et al., 2019). A key pathological driver of diabetic CVD is vascular remodeling, characterized by neointimal hyperplasia and accelerated atherosclerosis (Su et al., 2024). In diabetic patients undergoing percutaneous coronary intervention (PCI), chronic hyperglycemia exacerbates vascular injury by inducing oxidative stress, inflammation, and dysregulated VSMC proliferation. As a result, restenosis rates remain substantial despite advancements in stent design and drug-eluting technologies (Wilson et al., 2022; Ritsinger et al., 2015; Marfella et al., 2023).

Oxidative stress plays a central role in diabetic vasculopathy by promoting VSMC phenotypic switching, extracellular matrix deposition, and neointimal thickening (Sena et al., 2018). NOX4 is a crucial source of vascular reactive oxygen species (ROS), and its upregulation under hyperglycemic conditions accelerates vascular injury and atherosclerosis progression (Tang et al., 2023; Vendrov et al., 2024). Notably, redox and metabolic stress are closely intertwined in the vasculature, and AMPK has been reported to function as a key metabolic and redox sensor that can limit NADPH oxidase-derived ROS generation while supporting antioxidant defenses (Song and Zou, 2012). AMPK also integrates metabolic and redox signaling to restrain pathological VSMC proliferation and thereby contributes to vascular homeostasis (Wu and Zou, 2020). Inactivation of AMPK, with subsequent overactivation of mTOR and S6K1, has been strongly implicated in diabetic vascular remodeling (Al-Kuraishy et al., 2025; Lesniewski et al., 2017; Den et al., 2020).

Natural bioactive metabolites with both antioxidant and AMPK-activating properties have attracted considerable attention as adjunctive strategies for preventing diabetic vascular complications (Miao et al., 2023). Zingiber officinale (ginger) contains multiple polyphenolic metabolites with anti-inflammatory, antioxidant, and cardioprotective activities (Abdul Rani et al., 2025; Kiyama, 2020). Commonly studied ginger-derived metabolites, such as 6-gingerol and 6-shogaol, exert vasoprotective effects mainly through modulation of nuclear factor-kappa B (NF-κB) and mitogen-activated protein kinase (MAPK) signaling (Kiyama, 2020; Li et al., 2021; Li et al., 2017; Han et al., 2019). However, whether these classical ginger constituents robustly engage AMPK-dependent programs in VSMCs under hyperglycemic stress remains unclear. Gin A, a ginger-derived diarylheptanoid metabolite, exhibits unique pharmacological properties including metabolic pathway regulation and beneficial effects on lipid metabolism and adipogenesis. Prior studies suggest that these effects are associated with AMPK activation and the inhibition of S6K1 (Chen et al., 2018; Kim et al., 2018). Gin A has also been reported to attenuate monocyte-endothelial adhesion (Kim et al., 2018), supporting a potential vascular benefit. However, whether Gin A protects against diabetic vascular remodeling, and whether such protection is linked to modulation of oxidative stress and AMPK signaling with downstream mTOR/S6K1 pathway, has not been systematically investigated.

Accordingly, we investigated whether Gin A attenuates hyperglycemia-associated VSMC dysfunction and neointimal hyperplasia with particular focus on the contribution of AMPK signaling and redox regulation. We first examined the effects of Gin A in HG-stimulated A10 VSMCs by assessing proliferation, migration, and oxidative stress-related markers. AMPK dependency was tested in A10 VSMCs using pharmacological inhibition with Compound C, and the downstream mTOR/S6K1 signaling was evaluated in this setting. Complementary genetic evidence was obtained in primary HASMCs through siRNA-mediated knockdown of AMPKα. Iso-osmotic controls (L-glucose and D-mannitol) were examined in separate experiments to address the potential osmotic confounding. We further evaluated disease relevance in a streptozotocin/high-fat diet-induced diabetic rat carotid balloon-injury model by quantifying neointimal hyperplasia and examining *in vivo* AMPK activation and redox markers. Finally, we conducted preliminary oral pharmacokinetic profiling and a 14-day tolerability assessment in rats to inform translational feasibility.

## 2 Materials and methods

### 2.1 Materials

Gin A, streptozotocin (STZ), glucose, and Compound C were purchased from MedChemExpress (MCE, United States). The MTT reagent was obtained from Sigma (United States). Antibodies against NOX4 (NB110-58849, Novus Biologicals United States), PCNA (GTX100539, GeneTex, United States), AMPKα/p-AMPKα (#2532/#2531, Cell Signaling Technology, United States), mTOR/p-mTOR (GTX638220/GTX132803, GeneTex, United States), S6K1/p-S6K1(PA5-28597/PA5-17884, Invitrogen, United States), glyceraldehyde-3-phosphate dehydrogenase (GAPDH, #2118, Cell Signaling Technology, United States), histone H3 (H3, #9715, Cell Signaling Technology, United States) were used for Western blot. Assay kits for DHE (ID3560), MDA (BC0025), T-AOC (BC1315), SOD (BC5160), and BCA (PC0020) were obtained from Solarbio (Beijing, China). All serum and cell culture media were purchased from Procell Life Science and Technology Co., Ltd. Additionally, all organic solvents were purchased from Shanghai Life Science Company (Shanghai, China).

### 2.2 Cell culture

The VSMC line A10 (CRL-1476, ATCC, United States) was cultured in Dulbecco’s modified Eagle’s medium (DMEM, #30-2002, ATCC) supplemented with 10% fetal bovine serum (FBS, #30-2020, ATCC), 100 U/mL penicillin, and 100 μg/mL streptomycin. The cells were maintained at 37 °C in an incubator with 5% CO2. The culture medium was replaced every 48–72 h, and the cells were passaged upon reaching 80%–90% confluence. Primary HASMCs were purchased from Procell (Wuhan, China) and used between passages 2 and 4. Cells were cultured in complete medium (CM-H081; Procell) according to the manufacturer’s instructions at 37 °C in a humidified atmosphere of 5% CO2.

### 2.3 Cell proliferation assay

A10 cells were seeded into a 96-well plate at a density of 5 × 10^3 cells per well. After 24 h, the culture medium was replaced with fresh medium containing glucose at the indicated concentrations (10, 15, 30 and 50 mM) and Gin A at 0.1, 1, 10, or 100 μM. Gin A was dissolved in dimethyl sulfoxide (DMSO); an equivalent volume of DMSO was added to the control and glucose-treated groups as vehicle. After an additional 24 h of incubation, MTT reagent (0.5 mg/mL) was added, and the plates were incubated for 4 h at 37 °C. The supernatant was then carefully removed, and 100 μL DMSO was added to each well to dissolve the formazan crystals. Absorbance was measured at 490 nm using a microplate reader. Additionally, cell counts were performed using an automated cell counter. PCNA immunofluorescence staining was used solely for qualitative visualization of proliferating cells. Representative images from these experiments are shown for illustration only; no quantitative statistical analysis of PCNA immunofluorescence intensity was performed. Quantitative assessment of PCNA expression was carried out by Western blotting, as described in the protein extraction and Western blot analysis section.

### 2.4 Cell migration assay

For the wound-healing assay, A10 cells were cultured in a 6-well plate until they reached full confluence. A straight scratch was made in the monolayer using a 100-μL pipette tip. After washing with phosphate-buffered saline (PBS) to remove detached cells, the medium was replaced with 30 mM high-glucose (HG) medium containing 10 μM Gin A. Images were captured at 0 and 24 h, and the migration distance was quantified using ImageJ software. For the Transwell migration assay, Transwell inserts with an 8-μm pore size were placed in 24-well plates. A single-cell suspension of A10 cells (1 × 10^6 cells per well) was added to the upper chamber in serum-free medium. The lower chamber contained serum-containing medium with the indicated treatments to serve as a chemoattractant. The plates were incubated in a cell culture incubator for 24 h. After incubation, cells on the upper surface of the membrane were gently removed with a cotton swab. Cells that had migrated to the lower surface were fixed with 4% paraformaldehyde and stained with crystal violet. The membrane was then removed and examined under a microscope. Migrated cells were counted in five random fields per insert, and the mean value per insert was used for statistical analysis.

### 2.5 Intracellular assessment of oxidative stress

A10 cells were seeded onto coverslips in a 6-well plate and treated with HG (30 mM) medium. Gin A (10 μM) was added, and the cells were incubated for 24 h. The DHE probe (10 μM) was subsequently added, and the cells were incubated at 37 °C for 30 min. The ROS levels were assessed using confocal microscopy, and the fluorescence intensity was quantified with ImageJ software.

A10 cells were subjected to the indicated treatments, washed twice with ice-cold PBS and scraped into lysis buffer on ice. Cell suspensions were sonicated or homogenized and centrifuged at 12,000 × g for 10 min at 4 °C to remove debris. The resulting supernatants were used for MDA, T-AOC and SOD measurements.

### 2.6 Protein extraction and Western blot analysis

Cellular proteins were extracted using RIPA buffer containing protease and phosphatase inhibitors. The protein concentration was quantified with a BCA protein assay kit. The proteins were then separated by SDS-PAGE and transferred onto a PVDF membrane. The membrane was incubated with specific primary antibodies, followed by incubation with the corresponding secondary antibodies. The blots were subsequently imaged using a LI-COR scanner, and the grayscale values were analyzed using ImageJ software. For *in vivo* western blots, each lane corresponded to tissue from a single rat. Original blots are shown in Supplementary Figures S1–S3.

### 2.7 AMPK dependency assays

AMPK dependency was assessed using complementary pharmacological and genetic approaches. AMPK activity was inhibited with Compound C in A10 VSMCs, and AMPKα was knocked down by siRNA in primary HASMCs. We focused on AMPKα because phosphorylation at Thr172 is an established indicator of AMPK activation (Herzig and Shaw, 2018; Burkewitz et al., 2014).

#### 2.7.1 Pharmacological inhibition of AMPK in A10 VSMCs

A10 cells were co-treated with Gin A and the pharmacological AMPK inhibitor Compound C (HY-13418A, MCE, United States) under HG (30 mM) stimulation. Cells were pre-incubated with Compound C (10 μM) for 1 h and then exposed to Gin A (10 μM) under HG (30 mM) condition. Cell proliferation, migration, and pathway activation were then assessed as described in Sections 2.3–2.6.

#### 2.7.2 siRNA-mediated knockdown of AMPK in primary HASMCs

Small interfering RNAs targeting human AMPKα (si-AMPKα sequences; sense: 5′-GAG CGA CUA UCA AAG ACA UTT-3’; antisense: 5′-AUG UCU UUG AUA GUC GCU CTT-3′) and a scrambled siRNA were purchased from RiboBio (Guangzhou, China). Transfections were performed using Lipofectamine™ 3000 (Invitrogen, Carlsbad, CA, United States) in Opti-MEM® I reduced-serum medium according to the manufacturer’s instructions. Briefly, siRNA (100 pmol per well) and Lipofectamine 3000 reagent were mixed in Opti-MEM, incubated for 20 min at room temperature, and then added to the cells for 6 h. Knockdown efficiency was confirmed by Western blotting for AMPKα protein expression.

When cells reached approximately 80%–90% confluence, they were serum-starved for 2 h and then exposed for 24 h to normal-glucose medium or HG (25 mM) medium with or without Gin A (10 μM). The experimental design included five conditions in total. Cells were cultured in normal glucose with scrambled siRNA, in HG with scrambled siRNA, in HG plus Gin A with scrambled siRNA, in HG plus Gin A with si-AMPKα, or in normal glucose with si-AMPKα. An equal volume of vehicle (0.1% DMSO) was added to the corresponding control wells. Cell proliferation was measured by MTT assay, and AMPKα activation was evaluated by Western blotting of AMPKα phosphorylation.

### 2.8 Osmotic controls in primary HASMCs

For osmotic control experiments, primary HASMCs were seeded into 96-well plates, allowed to adhere overnight, and serum-starved for 12 h. Cells were then exposed for 24 h to normal-glucose (5.5 mM) medium, HG (25 mM), D-mannitol (25 mM), or L-glucose (25 mM). D-mannitol and L-glucose were added to normal-glucose medium at concentrations that yielded an osmolarity comparable to the HG condition. Cell proliferation and viability were assessed by MTT assay.

### 2.9 Diabetic rat carotid artery balloon injury model

Healthy 8-week-old Specific Pathogen-Free (SPF) Sprague-Dawley (SD) rats were selected as experimental subjects for the study. Type 2 diabetes was induced using low dose injection of STZ (30 mg/kg) combined with high-fat diet, as described previously (Gong et al., 2019). The rats with fasting blood glucose concentrations greater than 16.7 mM were enrolled for the carotid balloon injury experiments. Eligible rats were randomly assigned to Sham, Vehicle, or Gin A groups using a computer-generated sequence. Cage mates were distributed across different groups to minimize cage effects. Dosing formulations were prepared in identical coded vials by an independent technician, and the personnel administering the doses were aware of only these codes. Outcome assessment was also conducted under blind conditions. Investigators performing the histology and morphometry for the intima-to-media (I/M) ratio, the biochemical assays for MDA and T-AOC, and the Western blot analysis for PCNA and p-AMPK/AMPK worked with coded samples and analyzed images and data from files identified solely by code. The surgeon could not be blinded to injury versus sham for technical reasons and did not participate in downstream measurements. Unblinding occurred only after data verification and completion of the primary analysis.

The rats were housed in a controlled environment under a 12-h light/dark cycle and had unrestricted access to food and water. All animal experiments were approved by the institutional ethics committee and conducted in accordance with relevant guidelines and regulations.

In diabetic rats, carotid artery injury was induced by inserting a balloon catheter (2F Fogarty) into the common carotid artery via the external carotid artery. The balloon was then inflated with saline to distend the vessel and denude the endothelium. It was gently withdrawn three times to complete the injury induction (Matsuoka et al., 2005). The Gin A treatment group received Gin A at a daily dose of 10 mg/kg body weight via oral gavage immediately following surgery. The vehicle group received the corresponding vehicle (DMSO-containing solution) by oral gavage in the same volume and schedule as the Gin A group. The sham group was treated with the same surgical exposure without balloon inflation or arterial injury, and then received the same volume of DMSO as the Gin A group. Two weeks post-surgery, the carotid arteries were morphologically analyzed to assess neointimal hyperplasia. The tissue sections were stained with hematoxylin and eosin (H&E) and analyzed by Western blot to evaluate pathological changes and PCNA expression.

### 2.10 Preliminary pharmacokinetic evaluation in rats

The *in vivo* pharmacokinetics of Gin A were evaluated in SPF SD rats (n = 6). Gin A (10 mg/kg) was freshly dissolved in DMSO-based vehicle and administered as a single oral dose by gavage. Blood samples (0.3–0.5 mL) were collected from the tail vein at 0 (pre-dose), 0.25, 0.5, 1, 2, 4, 6, 8, 10, 12, 24, and 48 h after dosing. Plasma Gin A concentrations were determined using a validated liquid chromatography-tandem mass spectrometry (LC-MS/MS) assay. Pharmacokinetic parameters, including maximum plasma concentration (C_max_), time to C_max_ (T_max_), area under the plasma concentration-time curve from 0 to 24 h and 0–48 h (AUC_0-24_, AUC_0-48_), terminal elimination rate constant (λ_z_), terminal half-life (t_½_), mean residence time from 0 to 48 h (MRT_0-48_), area under the curve from 0 to infinity (AUC_0-_ ∞ ), and mean residence time from 0 to infinity (MRT_0-_ ∞ ), were estimated by noncompartmental analysis in R. Linear and semi-logarithmic (log_10_-transformed) plasma concentration-time profiles were plotted in R for visualization.

### 2.11 Preliminary toxicological evaluation in rats

A blinded, randomized toxicology study was conducted to assess the *in vivo* safety and tolerability of Gin A. Healthy 8-week-old SPF SD rats (n = 6 per group) were randomly assigned to a Gin A group (10 mg/kg) or a vehicle group (DMSO-based vehicle, equal volume). Gin A and vehicle were prepared aseptically and administered once daily by oral gavage for 14 days. On day 15, rats were anesthetized with 2% isoflurane, and blood was collected from the abdominal aorta. Serum was separated by centrifugation (2,000 × g, 15 min, 4 °C) and analyzed for alanine aminotransferase (ALT), aspartate aminotransferase (AST), creatinine (CR), blood urea nitrogen (BUN), and creatine kinase (CK) on an automated analyzer by blinded staff. After blood sampling, rats were euthanized by exsanguination under deep anesthesia. The heart, liver, spleen, lungs, and kidneys were collected, fixed in 4% paraformaldehyde (4 °C, 24 h), and processed and stained with H&E. Two board-certified pathologists, blinded to treatment, graded lesions on a semi-quantitative 0–3 scale (0 = none, 1 = minimal, 2 = moderate, 3 = marked), and any discrepancies were resolved by consensus. Randomization codes were released only after all biochemical and histological assessments were completed.

### 2.12 Statistical analysis

All quantitative data were expressed as mean ± standard deviation (SD). Normality was assessed with the Shapiro-Wilk test and homogeneity of variance with Levene’s test. For comparisons between two independent groups, Student’s t*-*test was used when both assumptions were met; Welch’s t-test was applied for normally distributed data with unequal variances; and the Mann-Whitney *U* test was used for non-normal data. For comparisons among three or more groups, one-way ANOVA with Tukey’s *post hoc* test was performed when assumptions were satisfied; otherwise, the Kruskal-Wallis test with Dunn’s *post hoc* test was applied. Comparisons were restricted to predefined groups. Dose-response relationships across graded concentrations were evaluated using linear regression and Spearman’s rank correlation. Statistical significance was defined as a two-tailed *P* < 0.05. All analyses and data visualization were conducted using R software.

Results are presented with group means and SD. Individual data points are overlaid to illustrate within-group variability. Unless otherwise specified, n in the figure legends refers to the number of wells per group used for statistical analysis. For cell-based assays, one well was treated as the experimental unit. When multiple images/fields were acquired from the same well, measurements were averaged within that well before statistical testing. Cell-based experiments were repeated on at least three separate days with comparable results to confirm reproducibility; unless otherwise specified, statistical analyses were performed using well-level measurements as defined above. For *in vivo* experiments, n denotes the number of animals per group.

## 3 Results

### 3.1 Gin A inhibits HG-induced VSMC proliferation and migration

**FIGURE 1:**
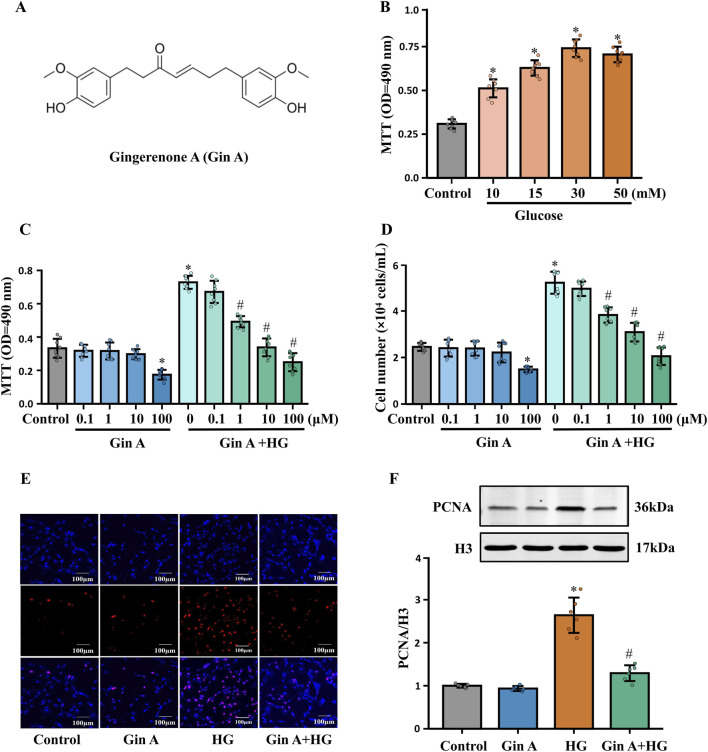
Gin A inhibits HG-induced proliferation of A10 cells **(A)** Structural formula of Gingerenone A (Gin A). **(B)** Effect of high glucose (HG) on the proliferation of A10 cells. A10 cells were incubated with 10-, 15-, 30-, or 50-mM glucose for 24 h. Cell proliferation was assessed by MTT assay (n = 8, *P < 0.05 vs. control). **(C,D)** Effect of Gin A on the proliferation of A10 cells. A10 cells were cultured for 24 h in normal medium or HG (30 mM) with Gin A at 0.1, 1, 10 or 100 μM. Cell proliferation was evaluated by MTT assay **(C)**, and automated cell counting **(D)** (n = 8, *P < 0.05 vs. control). **(E)** Representative PCNA immunofluorescence images of A10 cells cultured under control, HG (30 mM) and HG plus Gin A (10 μM) conditions for 24 h. Staining was repeated in three independent experiments with similar results; images are shown for qualitative illustration only and were not subjected to statistical analysis. **(F)** PCNA protein levels in A10 cells after 24 h of treatment with control medium, Gin A (10 μM), HG (30 mM) or HG plus Gin A (10 μM) for 24 h. Representative western blots and quantification of PCNA expression quantification of PCNA expression (normalized to H3) are shown (n = 6; *P < 0.05 vs. control; #*P* < 0.05 vs. HG alone).

**FIGURE 2:**
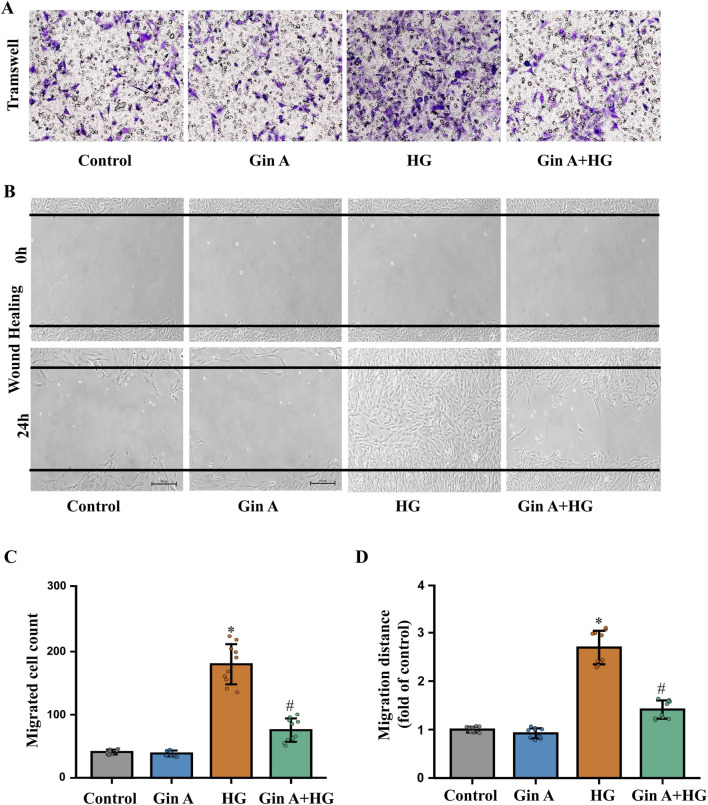
Gin A reduces HG-induced migration of A10 cells **(A)** Representative Transwell images showing A10 cell migration after 24 h of culture under control conditions, Gin A (10 μM), HG (30 mM) or HG plus Gin A (10 μM). **(B)** Representative wound-healing images at 0 and 24 h for A10 cells treated as in **(A)**. **(C)** Effect of Gin A on the increase in the number of migrating cells induced by HG (n = 10; *P < 0.05 vs. control; #*P* < 0.05 vs. HG alone). **(D)** Effect of Gin A on the increased migration distance induced by HG (n = 10; *P < 0.05 vs. control; #*P* < 0.05 vs. HG alone).

To investigate whether Gin A (chemical structure shown in Figure 1A) modulates these responses, A10 VSMCs were exposed to increasing concentrations of glucose (10, 15, 30 and 50 mM) for 24 h. The MTT assay showed a glucose concentration-dependent increase in VSMC proliferation (Figure 1B). 30 mM glucose was therefore selected to induce a hyperproliferative phenotype for subsequent experiments. Gin A was then tested at concentrations ranging from 0.1 to 100 μM. Gin A alone did not appreciably inhibit basal VSMC proliferation until 100 μM, but it significantly mitigated HG-induced proliferation in a concentration-dependent manner (Figures 1C,D). Consistently, PCNA immunofluorescence and immunoblotting showed that HG significantly increased PCNA expression, while Gin A reversed this increase (Figures 1E,F). In addition, scratch wound healing and Transwell assays demonstrated that Gin A (10 μM) significantly reduced the migratory capacity of A10 VSMCs under HG conditions (Figures 2A–D). These findings show that Gin A reduced HG-induced VSMC proliferation and migration.

### 3.2 Gin A inhibits HG-induced oxidative stress by suppressing NOX4 and restoring antioxidant capacity

**FIGURE 3:**
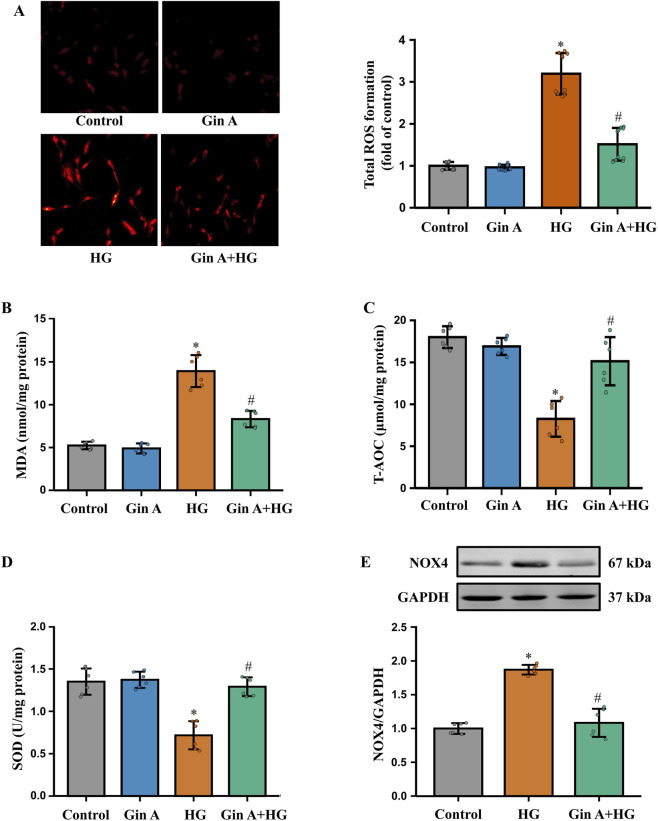
Gin A attenuates HG-induced oxidative stress in A10 cells **(A)** Intracellular ROS levels in A10 cells cultured for 24 h in control medium or HG (30 mM) in the absence or presence of Gin A (10 μM). ROS was evaluated by DHE staining and quantitative statistical data (n = 10; *P < 0.05 vs. control; #*P* < 0.05 vs. HG alone). **(B–D)** Oxidative stress-related indicators in A10 cells treated with control medium, Gin A alone (10 μM), HG (30 mM) or HG plus Gin A (10 μM) for 24 h. MDA **(B)**, T-AOC **(C)** and SOD activity **(D)** measured using assay kits (n = 6; *P < 0.05 vs. control; #*P* < 0.05 vs. HG alone). **(E)** NOX4 protein expression in A10 cells cultured for 24 h under control, HG or HG plus Gin A conditions. Representative western blots and NOX4/GAPDH ratios are shown (n = 6; *P < 0.05 vs. control; #*P* < 0.05 vs. HG alone).

We next investigated whether Gin A modulates HG-induced redox imbalance in A10 VSMCs by assessing intracellular ROS generation and oxidative stress-related biochemical markers. DHE staining showed that HG markedly increased superoxide-related fluorescence, whereas Gin A significantly reduced this signal (Figure 3A). HG also increased lipid peroxidation, reflected by elevated MDA levels, and Gin A significantly decreased MDA under HG conditions (Figure 3B). HG stimulation significantly decreased T-AOC and SOD levels, whereas co-treatment with Gin A partially reversed these changes (Figures 3C,D). Immunoblotting further showed that NOX4 protein expression was increased by HG and reduced by Gin A (Figure 3E). These results show that Gin A mitigates HG-induced redox imbalance in A10 VSMCs.

Because Gin A reduced oxidative stress and inhibited HG-induced proliferative and migratory responses, we next assessed whether Gin A modulates AMPK-centered signaling and its downstream mTOR/S6K1 axis.

### 3.3 Gin A activates AMPK and inhibits the mTOR/S6K1 pathway in A10 VSMCs

**FIGURE 4:**
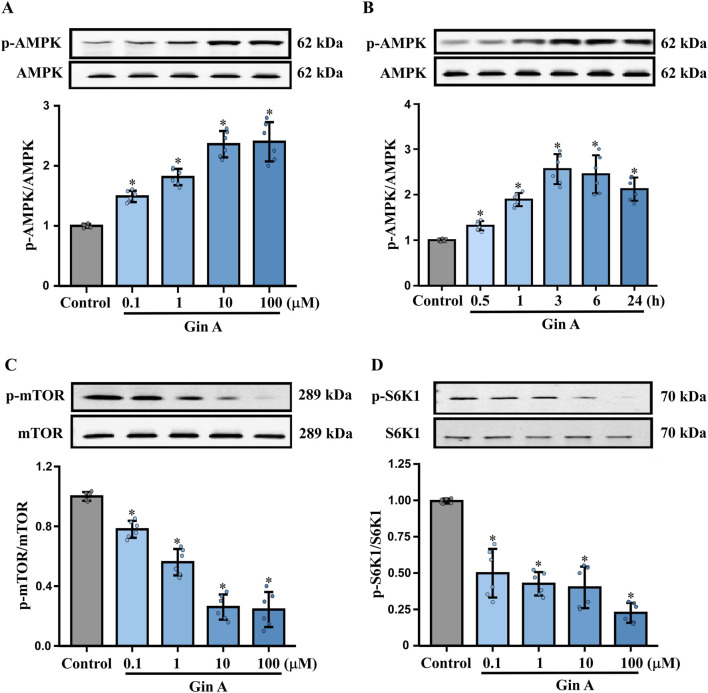
Gin A activates AMPK and suppresses mTOR/S6K1 signaling in A10 cells **(A)** Concentration-dependent effects of Gin A on AMPK phosphorylation. A10 cells were treated with Gin A at 0.1, 1, 10 or 100 μM for 24 h, and p-AMPK/AMPK ratios were determined by Western blot. **(B)** Time course of AMPK activation by Gin A. Cells were exposed to Gin A (10 μM) for 0.5, 1, 3, 6 or 24 h, and p-AMPK/AMPK levels were determined by Western blot. **(C)** Effects of Gin A on mTOR phosphorylation. A10 cells were treated with Gin A at 0.1, 1, 10 or 100 μM for 24 h, and p-mTOR/mTOR ratios were determined by Western blot. **(D)** Effects of Gin A on S6K1 phosphorylation. A10 cells were treated with Gin A at 0.1, 1, 10 or 100 μM for 24 h, and p-S6K1/S6K1 ratios determined by Western blot (n = 6; *P < 0.05 vs. control).

Building on the redox and functional effects observed under HG stimulation, we assessed AMPK activation and downstream mTOR/S6K1 pathway status in A10 VSMCs by Western blotting. Gin A increased AMPK phosphorylation (p-AMPK/AMPK) in a concentration-dependent manner (Figure 4A). Time-course experiments showed a rapid increase in p-AMPK that peaked at 3 h and remained above baseline at 24 h (Figure 4B). In parallel, Gin A reduced the p-mTOR/mTOR and p-S6K1/S6K1 ratios in a dose-dependent manner (Figures 4C,D).

**FIGURE 5:**
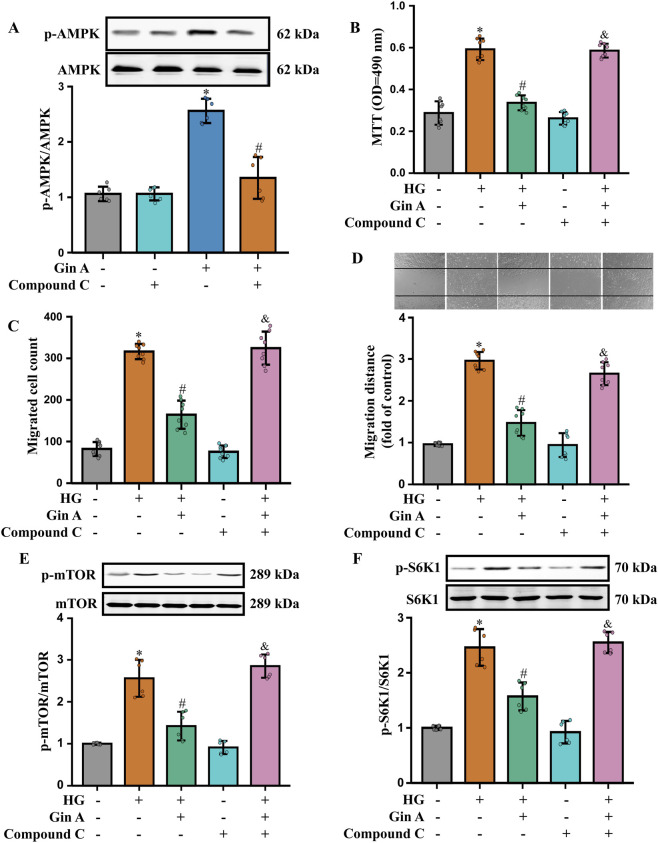
Role of AMPK activation in the Gin A-mediated inhibition of the proliferation and migration of HG-treated A10 cells **(A)** Effect of the AMPK inhibitor Compound C on Gin A-induced AMPK activation in HG-treated A10 cells. Cells were pre-incubated with Compound C for 1 h and then exposed to HG (30 mM) with or without Gin A (10 μM) for 24 h. Representative blots and p-AMPK/AMPK ratios are shown (n = 6; *P < 0.05 vs. control; #*P* < 0.05 vs. Gin A alone). **(B–D)** Effects of Compound C on the Gin A-induced inhibition of A10 cell proliferation and migration. Cell proliferation was determined by the MTT assay **(B)**. Cell migration was determined by Transwell **(C)** and wound healing **(D)** assays (n = 8; *P < 0.05 vs. control; #*P* < 0.05 vs. HG alone; &*P* < 0.05 vs. HG + Gin A). **(E,F)** Effects of Compound C on Gin A-mediated inhibition of mTOR/S6K1 signaling in HG-treated A10 cells. Representative blots and quantification of p-mTOR/mTOR **(E)** and p-S6K1/S6K1 **(F)** ratios are shown (n = 6; *P < 0.05 vs. control; #*P* < 0.05 vs. HG alone; &*P* < 0.05 vs. HG + Gin A).

The functional relevance of AMPK activation was then probed using the pharmacological AMPK inhibitor Compound C. We found that Compound C markedly attenuated the AMPK phosphorylation induced by Gin A (Figure 5A). The MTT assay, as well as the Transwell and wound-healing (scratch) assays, showed that Compound C attenuated the inhibitory effects of Gin A on the HG-induced proliferation and migration (Figures 5B–D). At the signaling level, Compound C also reversed the Gin A-associated reductions in p-mTOR/mTOR and p-S6K1/S6K1 (Figures 5E,F). These results indicate that Gin A modulates the AMPK/mTOR/S6K1 axis in HG-stimulated A10 VSMCs and that AMPK activity contributes to its protective effects on HG-induced VSMC dysfunction.

### 3.4 Primary HASMCs experiments support AMPKα dependency and address osmotic stress

**FIGURE 6:**
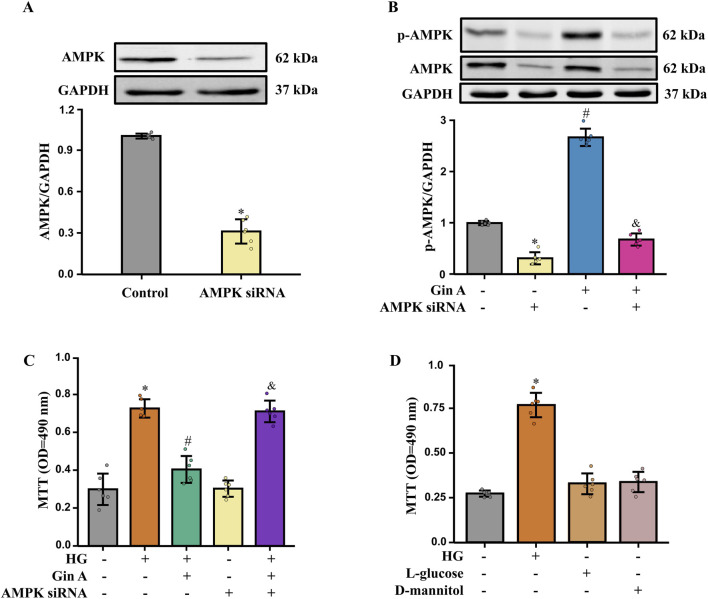
Experimental validation in primary HASMCs with siRNA knockdown and osmotic stress controls **(A)** Confirmation of AMPK knockdown in primary HASMCs. Representative western blots and AMPK/GAPDH ratios in scrambled siRNA- and AMPK siRNA-transfected cells are shown (n = 6; *P < 0.05 vs. control). **(B)** Effects of Gin A (10 μM) and AMPK knockdown on AMPK phosphorylation in HASMCs exposed to HG (25 mM) for 24 h. Representative blots and p-AMPK/GAPDH ratios are shown (n = 6; *P < 0.05 vs. control; #*P* < 0.05 vs. si-AMPK alone; &*P* < 0.05 vs. si-AMPK + Gin A). **(C)** Effects of Gin A and AMPK knockdown on HG-induced HASMC proliferation. Cells were exposed to normal glucose or HG (25 mM) with or without Gin A (10 μM) and with scrambled or AMPK-targeting siRNA, and proliferation was measured by MTT assay (n = 6; *P < 0.05 vs. normal-glucose control; #*P* < 0.05 vs. HG alone; &*P* < 0.05 vs. HG + Gin A with scrambled siRNA). **(D)** Osmotic control experiments in HASMCs. Cells were cultured for 24 h in normal glucose (5.5 mM), HG (25 mM), L-glucose (25 mM) or D-mannitol (25 mM), and proliferation was assessed by MTT assay (n = 6; *P < 0.05 vs. normal-glucose control).

Primary HASMCs were used to corroborate the observations in A10 VSMCs and to strengthen mechanistic inference with genetic evidence. Following transfection with AMPKα-targeting siRNA, immunoblotting showed a marked reduction in total AMPKα protein compared with scrambled controls, indicating effective knockdown (Figure 6A). Under HG (25 mM) conditions, Gin A increased the p-AMPK/GAPDH ratio in HASMCs, whereas this phosphorylation response was substantially attenuated in AMPKα-silenced cells (Figure 6B). Functionally, Gin A significantly reduced the HG-induced increase in MTT absorbance, while this antiproliferative effect was markedly weakened after AMPKα knockdown (Figure 6C). These results show that AMPKα knockdown blunted the antiproliferative effect of Gin A in primary HASMCs under HG stimulation.

Potential osmotic confounding was addressed in separate iso-osmotic control experiments in HASMCs. In MTT assays, HG (25 mM) significantly increased HASMC proliferation relative to normal-glucose medium, whereas iso-osmotic L-glucose or D-mannitol did not reproduce this proliferative effect (Figure 6D). This indicates that the proliferative response reflects HG stimulation rather than nonspecific osmotic effects.

### 3.5 Gin A attenuates neointimal hyperplasia and restores AMPK activation in diabetic rats

**FIGURE 7:**
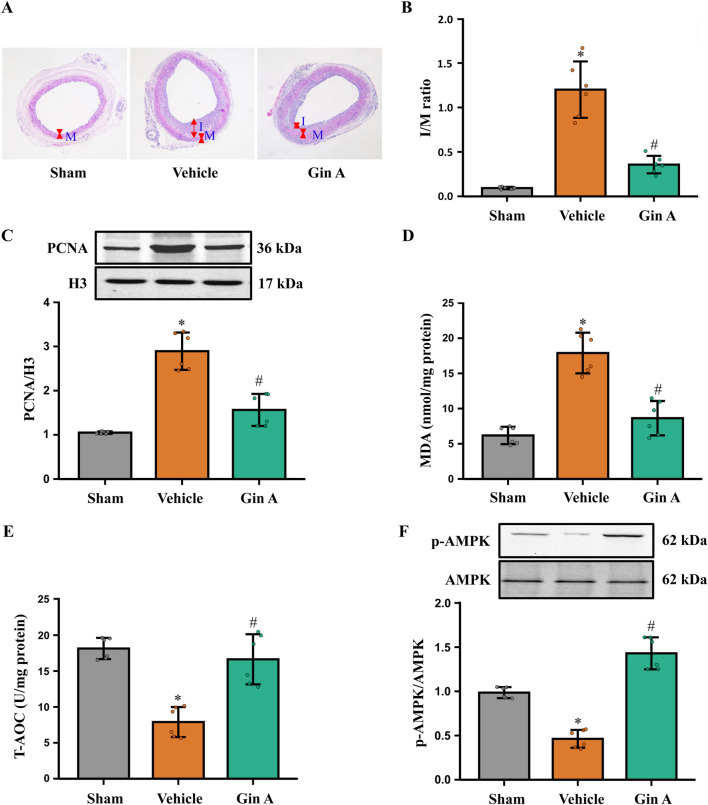
Gin A attenuates neointimal hyperplasia and restores AMPK activation in diabetic rats after carotid balloon injury **(A)** Representative H&E-stained cross-sections of carotid arteries from Sham, Vehicle and Gin A-treated diabetic rats 2 weeks after balloon injury (or sham operation). **(B)** Quantitative analysis of the intima-to-media (I/M) ratio in carotid arteries from the indicated groups. **(C)** Effects of Gin A on PCNA expression levels in carotid arteries of diabetic rats. Representative western blots and PCNA/H3 ratios are shown. **(D,E)** Effects of Gin A on oxidative stress markers in carotid arteries of diabetic rats. MDA **(D)** and T-AOC **(E)** measured in vascular tissue lysates. **(F)** Effects of Gin A on AMPK activation in carotid arteries of diabetic rats. Representative western blots and quantification of p-AMPK/AMPK ratios in carotid arteries are shown, indicating the restoration of AMPK activation by Gin A (n = 6; *P < 0.05 vs. Sham; #*P* < 0.05 vs. Vehicle).

Disease relevance was assessed in a diabetic rat carotid artery balloon-injury model. H&E staining showed that Gin A-treated rats developed a thinner neointima and had a significantly lower I/M ratio than vehicle-treated animals (Figures 7A,B). Immunoblotting for PCNA further showed that balloon injury markedly increased PCNA/H3 levels, whereas Gin A treatment significantly reduced these levels (Figure 7C). These results indicate that Gin A attenuates the injury-induced increase in vascular cell proliferation after carotid balloon injury. In injured arteries, MDA was reduced and T-AOC was increased in the Gin A group compared with vehicle-treated rats (Figures 7D,E). Gin A also increased the p-AMPK/AMPK ratio in the injured arteries compared with vehicle-treated rats (Figure 7F).

### 3.6 Preliminary pharmacokinetic evaluations of Gin A in rats

Systemic exposure after oral dosing of Gin A was assessed in rats. After a single oral dose of Gin A (10 mg/kg), plasma concentrations increased rapidly. The mean C_max_ was 0.0500 ± 0.0041 μg/mL and was reached at a mean T_max_ of 0.29 ± 0.10 h. Thereafter, Gin A concentrations declined gradually over the 48-h sampling period. The mean AUC_0-24_ and AUC_0-48_ were 0.2854 ± 0.0358 and 0.3714 ± 0.0508 μg h/mL, respectively. The terminal half-life was 12.41 ± 4.82 h, and the MRT_0-48_ was 12.83 ± 1.46 h (Table 1).

**TABLE 1:** Noncompartmental pharmacokinetic parameters of Gin A in rats.

Parameter	Mean ± SD
C_max_ (µg/mL)	0.0500 ± 0.0041
T_max_ (h)	0.29 ± 0.10
AUC_0-24_ (µg·h/mL)	0.2854 ± 0.0358
AUC_0-48_ (µg·h/mL)	0.3714 ± 0.0508
λ_z_ (1/h)	0.0608 ± 0.0156
t_½_ (h)	12.41 ± 4.82
MRT_0-48_ (h)	12.83 ± 1.46
AUC_0-_ ∞ (µg·h/mL)	0.4072 ± 0.0651
MRT_0-_ ∞ (h)	17.44 ± 6.35

C_max_: Maximum concentration.

T_max_: Time to reach maximum concentration.

AUC_0-24_: Area under the concentration-time curve from 0 to 24 h.

AUC_0-48_: Area under the concentration-time curve from 0 to 48 h.

λ_z_: Terminal elimination rate constant.

t_½_: Terminal half-life.

MRT_0-48_: Mean residence time from 0 to 48 h.

AUC_0-_ ∞ : Area under the concentration-time curve from 0 to infinity.

MRT_0-_ ∞ : Mean residence time from 0 to infinity.

**FIGURE 8:**
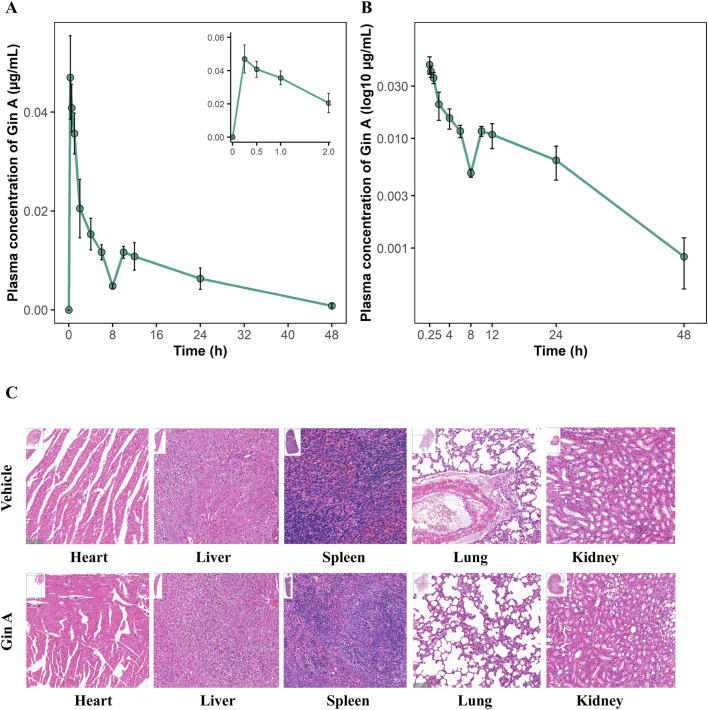
Preliminary pharmacokinetics and 14-day safety evaluation of Gin A in rats **(A)** Linear plasma concentration-time profile of Gin A following a single oral dose of 10 mg/kg in SD rats (n = 6). The inset highlights the early absorption phase (0–2 h) showing rapid attainment of C_max_. **(B)** Semi-logarithmic (log_10_-transformed) plasma concentration-time curve of Gin A over 48 h. The curve shows an approximately log-linear terminal phase consistent with first-order elimination. **(C)** Representative H&E-stained sections of heart, liver, spleen, lung and kidney from rats treated with vehicle or Gin A (10 mg/kg/day) for 14 days (n = 6). No treatment-related lesions, cellular degeneration, necrosis, inflammation or structural abnormalities were observed in any organ, and there were no discernible differences between Gin A-treated and vehicle-treated rats.

The plasma concentration-time curve is shown in Figure 8. The linear plot (Figure 8A) illustrates the rapid rise in plasma concentrations within the first 0.5 h, with the inset highlighting the early time window (0–2 h). The semi-logarithmic (log_10_-transformed) plot (Figure 8B) shows an approximately log-linear terminal phase.

### 3.7 Preliminary toxicological evaluations of Gin A in rats

Short-term tolerability of Gin A (10 mg/kg) was assessed in a 14-day repeat-dose study in healthy rats. Levels of ALT, AST, BUN, CR, and CK were comparable between the vehicle and Gin A groups after 14 days of treatment, with no statistically significant differences for any parameter (all *P* > 0.05; Table 2).

**TABLE 2:** Serum biochemical markers for toxicological evaluation of Gin A in rats.

Marker	Vehicle group	Gin A group	*P*
ALT (U/L)	70.31 ± 8.62	63.83 ± 6.83	0.181
AST (U/L)	182.56 ± 21.65	148.13 ± 44.67	0.132
BUN (mg/dL)	22.56 ± 1.95	21.68 ± 2.82	0.544
CR (μmol/L)	26.82 ± 6.77	24.28 ± 2.19	0.416
CK (U/L)	527.93 ± 77.22	466.90 ± 116.52	0.314

ALT, alanine aminotransferase; AST, aspartate aminotransferase; BUN, blood urea nitrogen; CR, creatinine; CK, creatine kinase.

Serum ALT, AST, BUN, CR, and CK, levels did not differ significantly between the vehicle and Gin A groups (all *P* > 0.05).

Histopathological assessment of major organs showed no apparent lesions in the heart, liver, spleen, lungs, and kidneys in Gin A-treated rats compared with vehicle controls (Figure 8C). No overt degeneration, necrosis, inflammatory infiltrates, or structural abnormalities were observed in either group. Under the conditions tested, no detectable organ toxicity was observed at 10 mg/kg of Gin A.

## 4 Discussion

Diabetes remains a major driver of macrovascular morbidity worldwide, and maladaptive vascular remodeling is a principal route through which chronic metabolic stress culminates in adverse cardiovascular outcomes (Sun et al., 2022; Ling et al., 2020; Dal Canto et al., 2019; Li et al., 2023). Despite contemporary drug-eluting stents and guideline-directed medical therapy, patients with diabetes who undergo revascularization continue to face an excess risk of restenosis, repeat revascularization, and late cardiovascular events (Ritsinger et al., 2015; Marx et al., 2023). These realities underscore the need for adjunctive strategies that do more than optimize procedural performance and instead directly temper the maladaptive vascular response to hyperglycemia (Li et al., 2023). In this study, Gin A, a ginger-derived metabolite, suppressed HG-associated VSMC proliferation and migration *in vitro* and reduced neointimal hyperplasia in a diabetic carotid balloon injury model *in vivo*. The convergence of cellular phenotypes, pathway perturbation, and *in vivo* injury outcomes points to Gin A as a modulator of remodeling biology in the diabetic setting.

A sustained redox imbalance is a defining feature of diabetic vasculopathy and contributes to pathological remodeling by perturbing redox-sensitive signaling and facilitating VSMC phenotypic switching (Sena et al., 2018; Li et al., 2023). Among enzymatic sources of vascular ROS, NADPH oxidases are particularly relevant to persistent oxidative stress in diabetes (Song and Zou, 2012; Kračun et al., 2025). In this context, NOX4 has been linked to diabetes-associated vascular injury and neointimal hyperplasia, consistent with ongoing ROS generation engaging growth- and migration-related programs (Tang et al., 2023; Deliyanti et al., 2020; Tong et al., 2015). In our study, HG exposure increased intracellular ROS production, elevated MDA levels, and upregulated NOX4 expression in A10 VSMCs, while reducing T-AOC and SOD activity. Gin A reversed these changes, indicating that preservation of redox homeostasis is a key feature of its protective action under hyperglycemic stress. The present work does not determine whether NOX4 downregulation represents a direct molecular action of Gin A or occurs secondary to upstream signaling changes. Nevertheless, the coupled reduction in ROS burden and recovery of endogenous antioxidant capacity indicates that Gin A counteracts a redox milieu characteristic of remodeling-prone VSMC states.

The signaling data further implicate AMPK-centered regulation as functionally important for Gin A-mediated suppression of remodeling-relevant VSMC behavior. AMPK integrates energetic cues with oxidative stress signaling and interfaces with growth pathways governing VSMC proliferation and migration (Herzig and Shaw, 2018; Rodríguez et al., 2021). Under diabetic conditions, reduced AMPK activity has been associated with heightened activity of growth-promoting cascades, including the mTOR/S6K1 axis, which is tightly linked to proliferative remodeling (Li et al., 2023; Saxton and Sabatini, 2017). In HG-stimulated VSMCs, Gin A increased AMPK phosphorylation while suppressing downstream mTOR and S6K1 activation. Two complementary perturbation approaches reinforce the inference that AMPK activation is necessary for a substantial component of Gin A’s effect. Pharmacological AMPK inhibition with Compound C attenuated Gin A-mediated suppression of HG-driven proliferation and migration and weakened its inhibitory effects on mTOR/S6K1 activation. Likewise, genetic knockdown of AMPKα in primary HASMCs reduced the antiproliferative effect of Gin A. Collectively, these observations support a mechanism in which Gin A engages AMPK-dependent signaling to restrain mTOR/S6K1-linked growth signaling and stabilize VSMC behavior under hyperglycemic challenge, accompanied by improvement in oxidative stress markers. This interpretation aligns with prior evidence that AMPK activation limits VSMC proliferation and migration and reduces injury-induced vascular remodeling (Stone et al., 2013), and with reports connecting AMPK to redox regulation in cardiovascular systems (Song and Zou, 2012; Rodríguez et al., 2021).

Interpretation of HG-based *in vitro* models also requires explicit control for hyperosmolarity. We therefore incorporated iso-osmotic controls using L-glucose and D-mannitol. The proliferative response elicited by D-glucose was not reproduced by these osmotic controls, supporting the conclusion that the phenotype reflects glucose-specific stimulation rather than a nonspecific osmotic stress response. This strengthens the biological specificity of the HG-triggered response interrogated here and reduces concern that Gin A primarily counteracts a generic stress adaptation.

These findings align with, and extend, existing work on Gin A and ginger-derived metabolites. Prior studies reported that Gin A inhibits S6K1 activity and improves insulin-related signaling, placing it within kinase networks central to cardiometabolic regulation (Chen et al., 2018; Suk et al., 2017). Gin A has also been shown to attenuate inflammatory endothelial activation through suppression of NF-κB signaling, suggesting engagement of stress-response pathways beyond a single cell type (Kim et al., 2018). While classical ginger constituents such as 6-gingerol and 6-shogaol are often discussed in relation to anti-inflammatory signaling (Abdul Rani et al., 2025; Kiyama, 2020; Li et al., 2021; Li et al., 2017; Han et al., 2019), our data connect Gin A more directly to remodeling-relevant pathways by linking AMPK activation to redox stabilization and suppression of mTOR/S6K1-associated VSMC activation. These results do not exclude contributions from parallel pathways. Rather, they identify AMPK/mTOR/S6K1 as a central axis sufficient to account for a substantial component of the observed vascular protection in the tested models.

From a translational standpoint, targeting remodeling biology is particularly attractive in diabetes, where technically successful revascularization does not fully eliminate residual vascular risk (Ritsinger et al., 2015). In our study, oral dosing yielded measurable systemic exposure with a terminal half-life compatible with once-daily administration. Additionally, a 14-day repeat-dose regimen showed no overt biochemical or histopathological toxicity in major organs under the tested conditions. These findings are necessarily preliminary but support further dose-ranging studies and longer-duration safety assessments. Practical translation will also require a clearer understanding of metabolic liabilities and potential drug-drug interactions, especially in view of diabetes-associated polypharmacy (Marx et al., 2023). The absence of CYP interaction profiling is therefore an important limitation of the current work. In addition, the diabetic balloon injury model is well suited to interrogate neointimal hyperplasia and has precedent in diabetic vascular intervention studies (Gong et al., 2019; Matsuoka et al., 2005). However, it does not fully recapitulate the complexity of human diabetic vascular disease, including advanced atherosclerotic plaque biology and endothelial dysfunction. Validation across additional vascular beds and disease-relevant models will be essential before extending conclusions beyond restenosis-like remodeling. It will also be important to explore strategies to improve Gin A exposure, such as rational structural optimization or nanocarrier-based delivery systems, which have been proposed for other natural products and for Gin A-derived analogues (Moa et al., 2025; Huang et al., 2024).

In summary, this study shows that Gin A attenuates diabetic vascular remodeling by suppressing oxidative stress and limiting pathological VSMC proliferation and migration through an AMPK-dependent mechanism converging on the mTOR/S6K1 axis. The agreement across rodent and human VSMC systems, pathway perturbation experiments, and *in vivo* neointimal hyperplasia outcomes provides a coherent preclinical basis for continued investigation. Future work should clarify upstream events leading to AMPK activation, define the determinants of NOX4 regulation, and establish longer-term exposure-response relationships and safety margins needed for potential clinical translation.

## Data Availability

The original contributions presented in the study are included in the article/Supplementary Material, further inquiries can be directed to the corresponding authors.

## Glossary

ALT: Alanine aminotransferase
AMPK: AMP-activated protein kinase
AST: Aspartate aminotransferase
AUC: Area under the concentration-time curve
BCA: Bicinchoninic acid
BUN: Blood urea nitrogen
CK: Creatine kinase
**C**_**max**_: Maximum plasma concentration
CR: Creatinine
CVD: Cardiovascular disease
DHE: Dihydroethidium
DM: Diabetes mellitus
DMSO: Dimethyl sulfoxide
FBS: Fetal bovine serum
GAPDH: Glyceraldehyde-3-phosphate dehydrogenase
Gin A: Gingerenone A
HASMCs: Human aortic smooth muscle cells
H&E: Hematoxylin and eosin
HG: High glucose
I/M: Intima-to-media
MAPK: Mitogen-activated protein kinase
MDA: Malondialdehyde
mTOR: Mechanistic target of rapamycin
MRT: Mean residence time
MTT: Methyl thiazolyl tetrazolium
NF-κB: Nuclear factor-kappa B
NOX4: NADPH oxidase 4
PCNA: Proliferating cell nuclear antigen
PCI: Percutaneous coronary intervention
ROS: Reactive oxygen species
SD: Standard deviation
SOD: Superoxide dismutase
S6K1: p70 ribosomal S6 kinase 1
SPF: Specific pathogen-free
STZ: Streptozotocin
T-AOC: Total antioxidant capacity
**T**_**max**_: Time to maximum plasma concentration
VSMCs: Vascular smooth muscle cells
