# Supplementary figures and images for: Gingerenone A attenuates diabetic vascular remodeling through AMPK/mTOR/S6K1 signaling
Source: Front Pharmacol. 2026 Jan 21;17:1706103. doi: 10.3389/fphar.2026.1706103 (PMC12868287; doi:10.3389/fphar.2026.1706103)

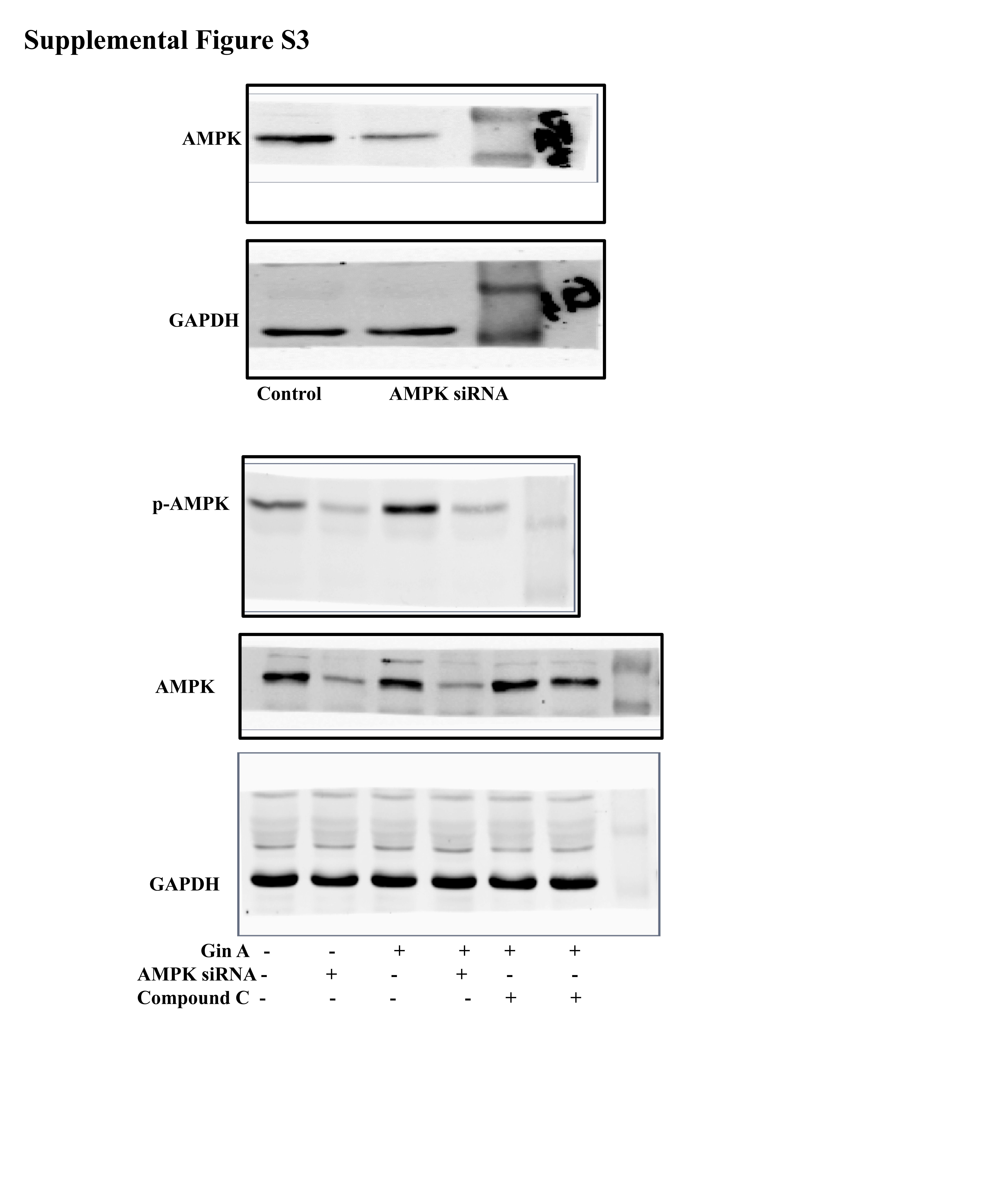

Supplement: Supplementary file 1 [file Image3.jpeg]

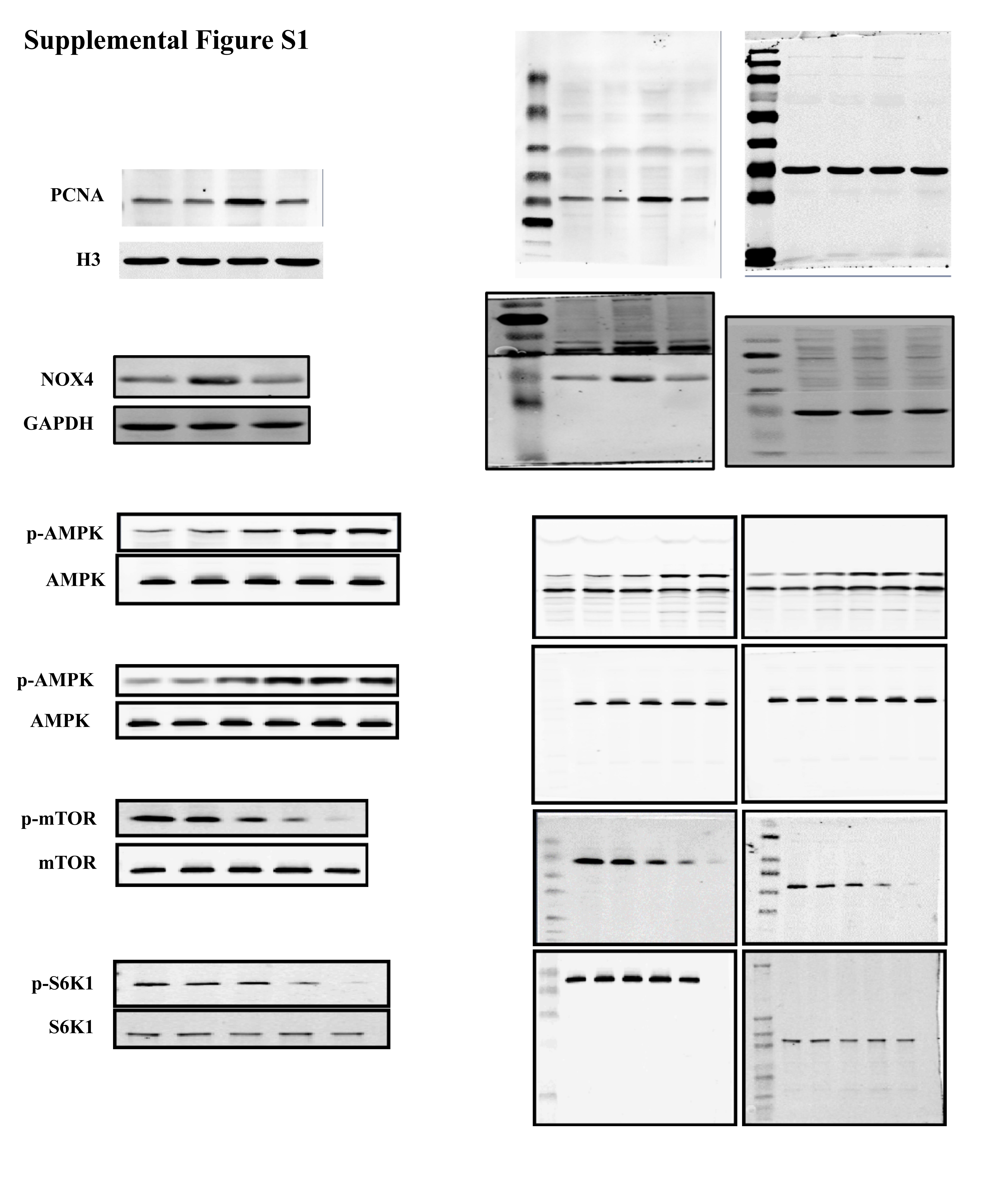

Supplement: Supplementary file 2 [file Image1.jpeg]

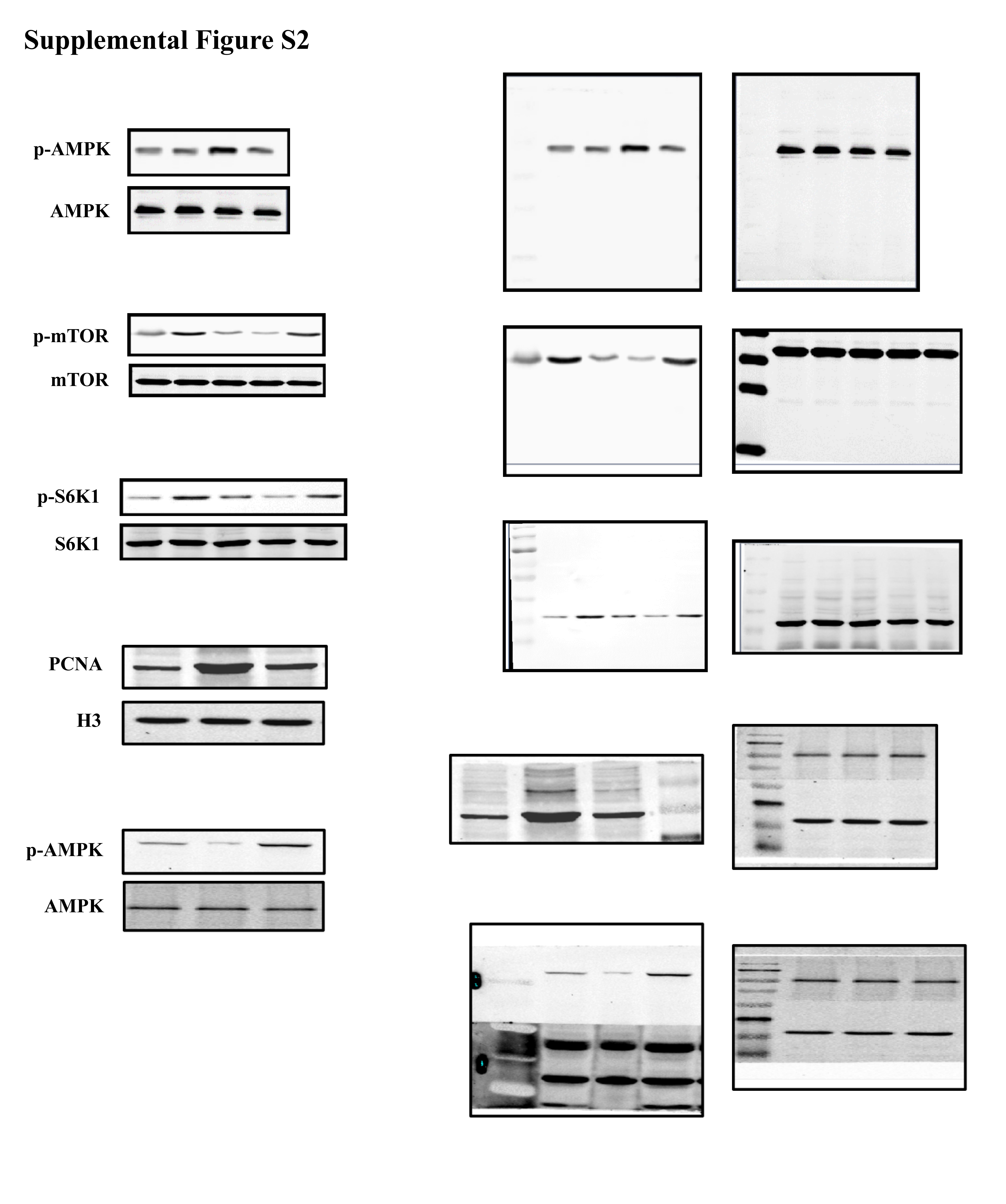

Supplement: Supplementary file 3 [file Image2.jpeg]
